# Shared decision-making in neurosurgery: a scoping review

**DOI:** 10.1007/s00701-021-04867-3

**Published:** 2021-05-03

**Authors:** Alba Corell, Annie Guo, Tomás Gómez Vecchio, Anneli Ozanne, Asgeir S. Jakola

**Affiliations:** 1 Department of Neurosurgery, Sahlgrenska University Hospital, Blå stråket 5, 41345 Gothenburg, Sweden; 2 Institute of Neuroscience and Physiology, Department of Clinical Neuroscience, University of Gothenburg, Sahlgrenska Academy, Gothenburg, Sweden; 3 Institute of Health and Care Sciences, Sahlgrenska Academy, University of Gothenburg, Gothenburg, Sweden; 4 Department of Neurology, Sahlgrenska University Hospital, Gothenburg, Sweden; 5 Department of Neurosurgery, St.Olavs University Hospital, Trondheim, Norway

**Keywords:** Decision-making, Shared, Neurosurgery, Surgical oncology, Spine, Patient-centered care, Decision aids

## Abstract

**Background:**

In modern neurosurgery, there are often several treatment alternatives, with different risks and benefits. Shared decision-making (SDM) has gained interest during the last decade, although SDM in the neurosurgical field is not widely studied. Therefore, the aim of this scoping review was to present the current landscape of SDM in neurosurgery.

**Methods:**

A literature review was carried out in PubMed and Scopus. We used a search strategy based on keywords used in existing literature on SDM in neurosurgery. Full-text, peer-reviewed articles published from 2000 up to the search date February 16, 2021, with patients 18 years and older were included if articles evaluated SDM in neurosurgery from the patient’s perspective.

**Results:**

We identified 22 articles whereof 7 covered vestibular schwannomas, 7 covered spinal surgery, and 4 covered gliomas. The other topics were brain metastases, benign brain lesions, Parkinson’s disease and evaluation of neurosurgical care. Different methods were used, with majority using forms, questionnaires, or interviews. Effects of SDM interventions were studied in 6 articles; the remaining articles explored factors influencing patients’ decisions or discussed SDM aids.

**Conclusion:**

SDM is a tool to involve patients in the decision-making process and considers patients’ preferences and what the patients find important. This scoping review illustrates the relative lack of SDM in the neurosurgical literature. Even though results indicate potential benefit of SDM, the extent of influence on treatment, outcome, and patient’s satisfaction is still unknown. Finally, the use of decision aids may be a meaningful contribution to the SDM process.

**Supplementary Information:**

The online version contains supplementary material available at 10.1007/s00701-021-04867-3.

## Introduction

Advances in the medical field during the last decades have led to a range of available options for use in the decision-making process [36]. The development of the healthcare system as a whole has shifted toward a higher degree of person-centered care, which incorporates patients and their values, needs, and preferences [12, 40]. The tool of shared decision-making (SDM) in clinical practice has gained interest mainly during the last decade. SDM aims to include the patient to a larger extent in decision-making regarding the next treatment step [3]. The use of SDM overall in the medical field has significantly increased over the years, from between 1 and 50 publications per year between 1968 and 1994, to numbers in the thousands during the most recent years [7].

While an informed consent is based on presenting information to the patient by the physician, SDM includes the patient and the process is based on mutual respect and participation in the discussion [8]. There is, however, no clear definition of SDM [26] but generally SDM can be identified through four steps: first, the patient is informed of the need for a decision regarding a health issue and the patient’s own thoughts are important. Secondly, the process continues with a presentation of the pros and cons with the different options by the healthcare provider, followed by the third step which is a discussion led by the professional to support the patient in the thought process in an informative way and, lastly, in the fourth step, the patients wish to decide is discussed and they either make decision or defer it [49]. The discussion should also lift the possible complications and management of these, so the patient can fully grasp the associated risks with treatment option. The logic behind the core SDM model is that what the physician deems relevant may differ from what is considered important by a patient capable to decide. Across multiple scenarios, SDM strives to integrate the best available clinical evidence with the patient’s values and preferences [14, 30, 47].

A large systematic review of SDM in the field of surgery showed that 29.3% of patients and 43.6% surgeons experienced that their consultation was performed in a SDM fashion, illustrating the discrepancy between perception of what SDM is and in what manner the consultation was performed [10]. The experience of lack of information is not uncommon, and one way to improve this may perhaps be to actively include the patient in decision-making regarding their own care [17, 21, 44, 56].

Neurosurgery is considered a high-risk surgical field [9, 41]. Moreover, asymptomatic or minimally symptomatic lesions are nowadays more often encountered in clinical practice due to increased availability of radiologic diagnostics and a generally older population [33, 37, 45, 46]. It is not always a decision on whether to treat or not, but significantly different treatment alternatives may be relevant (e.g., endovascular treatment versus clipping for intracranial aneurysms or radiosurgery versus resection for vestibular schwannomas). Thus, the risk–benefit profile in association with the various options requires a deeper patient involvement in the decision-making, and it can be considered to be our responsibility as professionals to discuss the different alternatives where they exist. Furthermore, many patients seem to prefer SDM regarding medical decisions; however, some patients with brain tumors may suffer from cognitive impairment and be unable to make the decision by themselves and would benefit from support from relatives [7, 17, 18, 20].

Even though the awareness regarding SDM is increasing, it is not widely incorporated in clinical practice [24]. In neurosurgery, we expected the SDM literature to be limited. The aim of this scoping review was to evaluate the current status of the literature regarding SDM in neurosurgery.

## Methods

### Design — literature review

A literature review was performed in order to present the existing literature on SDM in neurosurgery and to explore the main themes.

### Search strategy

The literature search was performed using the databases PubMed and Scopus on February 16, 2021. It was performed by a trained librarian, assisted by the review authors (AC, AG). Selection of database was based on the area of the question. The search strategy consisted of two blocks: neurosurgery and shared decision-making. To directly select keywords related to the topic of interest, we included MeSH (Medical Subject Headings) terms of the National Library of Medicine to identify relevant articles in PubMed as well as relevant keywords and synonyms. Additionally, corresponding search terms were used in the literature search performed in Scopus. The search strategy was based on keywords used in existing literature of shared decision-making in neurosurgery. It included articles published from 2000 up to the search date February 16, 2021. A detailed description of the used search strategy is presented in Supplementary Tables 1 and 2. To identify any additional relevant articles, references of all articles selected for reviewing in full text were examined. A PRISMA flowchart was created [27].

**Table 1:** Characteristics of included studies screened through database (*N* = 14)

Study author	Condition/topic	Study design	Objective/aim	Participants	Main finding
Barrett et al. (2002)	Lumbar herniated disk	Prospective, observational study with questionnaires	A study to evaluate impact of SDM aids (video program) on the shared decision-making in patients with lumbar herniated disk	*N* = 231	There was an increased preference for laminectomy after viewing the video program, although for reasons unknown
Cheung et al. (2010)	Vestibular schwannoma	Prospective, conjoint surveys, and demographics questionnaires	A study to demonstrate methodology and feasibility of adapting conjoint analysis for mapping clinical outcomes expectations to treatment decisions in vestibular schwannoma management	*N* = 196	Treatment decisions for a synthetic clinical scenario revealed different drivers of choice selection among the study cohorts. Main findings were that all three cohorts preferred GK radiosurgery despite a highly pessimistic risk level for cancer. Furthermore, younger and older prospective patients have indistinguishable preference profiles, suggesting that age should not be used as a factor to exclude particular treatment choices from consideration
Graham et al. (2018)	Vestibular schwannoma	Prospective cohort study, using decisional conflict scale (DCS) form, after consultation	Investigate decisional conflict in patients with vestibular schwannoma deciding between surgery or non-surgical management	*N* = 77	A decisional conflict was experienced in 20% of the patients. Involving patients in decision-making reduced the degree of uncertainty
LaHue et al. (2017)	Parkinson’s disease	Structured interview, preoperative and up to 4 years after surgery	Investigate factors that influenced patients to choose one DBS method over the other (sleep vs awake)	*N* = 89	Patients satisfied with both methods; identification of preferences optimizes patient experience and satisfaction
Lucchiari et al. (2010)	Glioma	Quantitative study, follow-up, and questionnaires after first surgery and at 3 months follow-up	Investigate if quality of life (QoL) is linked to unmet needs in formation management and decision involvement. Investigation of degree of involvement in clinical process	*N* = 84	Patients satisfied with decisional involvement might be able to better cope with disease. The need for better understanding of patient preferences related to information and decision sharing
Moshtaghi et al. (2018)	Vestibular schwannoma	Survey though Acoustic Neuroma Association (ANA), retrospective, with vestibular schwannoma	Assessment of decision-making process of patients with vestibular schwannoma	*N* = 789	The authors found that 80% of the patients visited multiple vestibular schwannoma specialists. The number of neurootologists consulted correlated with higher decision satisfaction
Müller et al. (2010)	Vestibular schwannoma	Postal questionnaire survey	Investigation of decision-making of patient and therapy decision	*N* = 739	In the cohort, 69% only received information regarding one treatment option, mainly surgery, and usually not enough information regarding side effects of the treatments
Murthy et al. (2016)	Spinal stenosis	Case report, evaluation preoperative and postoperative	Illustrative case report regarding facilitating shared decision-making in older patients	*N* = 1	A multidisciplinary approach and in-depth discussion with the patients regarding risks and benefits facilitated a shared decision-making in this case
Neve et al. (2020)	Vestibular schwannoma	Qualitative inductive thematic analysis, semi-structured interviews after consultation	Aim to identify factors that influence a patient’s decision for a certain treatment strategy	*N* = 18	Medical and patient-related themes were identified, which will help physicians to engage the patient in shared decision-making
Prasad et al. (2018)	Vestibular schwannoma	Quantitative study, statistical analysis clinical journal-based	A study to analyze long-term outcomes of wait-and-scan in the treatment of patients with VS, including factors contributing to decision-making	*N* = 576	It was the patients’ decision to proceed with the wait-and-scan approach after a discussion over risks and benefits for the management of VS. The successes of wait-and-scan can be very high. Moreover, the wait-and-scan modality is an optimal strategy in elderly patients with unilateral solitary vestibular schwannoma
Thorne et al. (2002)	Neurosurgical care	Postal questionnaires up to 1 year after discharge	Investigate patient satisfaction with neurosurgical services	540 questionnaires sent, *N* = 364 returned	A majority (84%) felt they were involved as much as they wished in decision-making process. High patient satisfaction with the neurosurgical practice, dissatisfaction with administrative arrangements
Van de Belt et al. (2018)	Glioma	Exploratory study with 3D printed models of glioma, semi-structured interviews before and after consultation	Use of an actual-size 3D model of glioma based on patients’ MRI examination to facilitate high-quality information to patients with glioma	*N* = 11	The 3D printed model showed promise as a tool to include patients in the decision-making process and for patients to better understand situation, possible treatment strategies, and risks
Weernink et al. (2016)	Parkinson’s disease	Patient interviews and questionnaires	A study to elicit patient preferences regarding the main treatments in Parkinson’s disease, including patients preferred and perceived involvement in decision-making about treatment	*N* = 192 (for preferences of main treatments)/*N* = 212 (for perceived involvement)	There is no “one-size-fits-all” approach to choosing treatments; benefits and side effects of treatment have different importance for patients. Many patients preferred an active role in decision-making about treatment
Zeng et al. (2017)	Brain metastases	Prospective inclusion, debriefing questionnaire after consultation	Aim to identify patient preferences in decision-making regarding non-resectable brain metastases: passive role or active role with either stereotactic radiosurgery (SRS) alone or with whole-brain radiation therapy (WBRT)	*N* = 23	Most patients take an active role if information is presented adequately. Most preferred SRS alone. Quality of life, functional independence, and influence of treatment on survival is important

**Table 2:** Articles identified through references of articles reviewed (*N* = 8)

Study author	Condition/topic	Study design	Objective/aim	Participants	Main finding
Andersen et al. (2019)	Lumbar herniated disc	Prospective development of decisional aid, structured interview, simulation of consultation	To evaluate the need of SDM in a spine surgery clinic and development of a decisional aid (PtDA) including testing it	*N* = 39	An SDM approach was not extensively practiced in the clinic and the tool PtDA developed was usable for both patients and practitioners
Brennum et al. (2015)	Glioma	Semi-structured focus group interviews	Interviews with focus to investigate attitude toward more extensive surgery with a trade-off between neurological deficit and survival time	*N* = 8 patients, *N* = 7 experts	Large variance in definition of quality of life, and degree of risk of neurological deficit. Individual assessments necessary, and patients with well-informed decisions may even later regret it
Diaz et al. (2009)	High-grade glioma	Transversal, descriptive and correlational study, use of Hospital Anxiety and Depression Scale questionnaire (HADS) at time of discharge	A study to investigate relationship between quality of information during the surgical decision-making discussion and anxiety level in patients with high-grade glioma	*N* = 26	Those patients with wish to be informed regarding their condition had a lower level of anxiety, as well as in those with higher degree of understanding the information and level of satisfaction
Kim et al. (2015)	Lumbar stenosis	Prospective, follow-up questionnaires	Aim to investigate factors that influence surgical decision-making for treatment of lumbar spinal stenosis with use of preference-based shared decision-making process	*N* = 555	With the preference-based shared decision-making process, factors leading to a surgical decision included motor weakness, male gender and amount of visible disability
Nellis et al. (2017)	Vestibular schwannoma	Prospective data collection	Aim to investigate the decision between surgical resection or active surveillance in patients with acoustic neuroma	*N* = 216	Factors such as quality of life, depression, or self-esteem did not seem to influence the decision-making. Younger patients with larger and growing tumors and symptoms such as hearing loss and worse headaches were more likely to select surgical resection, possibly due to longer life expectancy compared with elderly patients even though the surgery is associated with risks
Roszell et al. (2016)	Spinal stenosis	Prospective inclusion, telephone follow-up for up to 3 years	Studying factors influencing patients to undergo spine surgery, not including patients with additional disabling conditions	*N* = 39 of which *N* = 20 underwent surgery	The surgical decision-making was influenced more by the issues related to health and quality of life, rather than other factors such as pain or disability
Rozmovits et al. (2010)	Benign brain lesion	Semi-structured interviews	Aim to identify the need of information in patients who previously received neurosurgical care for benign brain tumor, arteriovenous malformation or unruptured aneurysm	*N* = 25	Patients require improved communication, and the wants and needs of information vary between patients
Weiner et al. (2006)	Spinal conditions	Prospective questionnaires following, and independent for, the visit with the attending surgeon	Study to investigate patients’ preferences with regards to physician and the patient’s role in the surgical decision-making, in addition to possible ethical consideration	*N* = 192	Their findings showed that patients in need of spinal surgery often want the surgeon to make the decision, and in more complex cases, the patient should be fully informed to help the patient make an informed decision

### Eligibility criteria

**Fig. 1:**
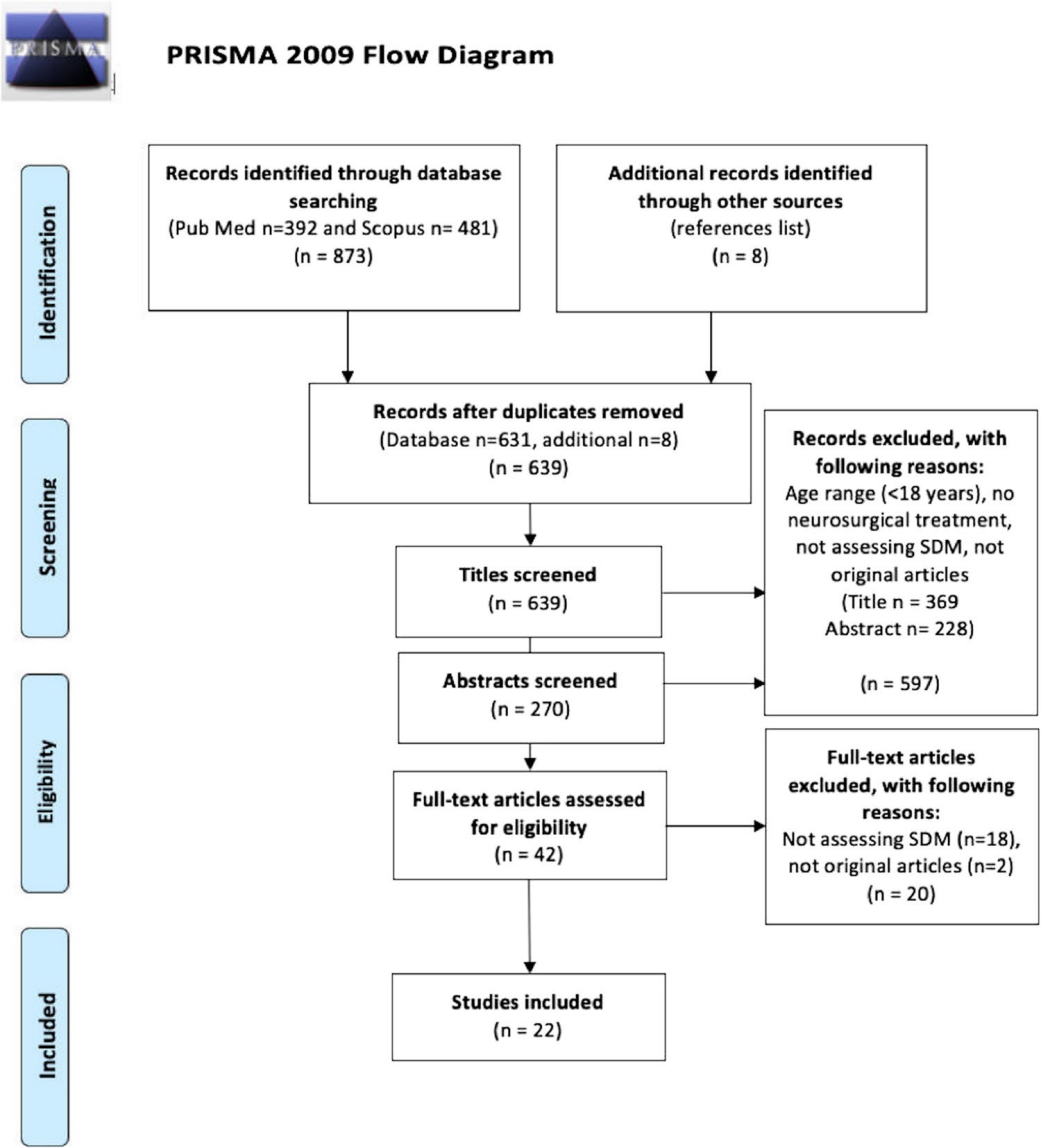
PRISMA flowchart

Eligible criteria were prospective and retrospective original full-text available, peer-reviewed articles published between from 2000 up to the search date February 16, 2021, patients 18 years and older, and articles regarding shared decision-making in neurosurgery. Exclusion criteria were SDM from other perspectives than patients and articles written in other languages than English or the Scandinavian languages (See the PRISMA flowchart for article inclusion (Fig. 1)).

All articles identified by database search were screened based on information in titles and abstracts. Articles selected during the screening were reviewed in full text by three review authors separately (AC, AG, TGV) and the discordant articles (*N* = 3) were reviewed by a senior consultant in neurosurgery (ASJ).

### Analysis and synthesis of results

Included articles were collated and summarized for reporting results. No meta-analysis was planned as a small sample with large heterogeneity was anticipated. The study was planned to only be descriptive in character. The articles were further analyzed using a thematic analysis grid. We aimed primarily to identify the patient groups included in SDM processes, the methods used to plan or assess SDM interventions, the type of decision topics addressed by SDM interventions, and the most relevant findings on the field of neurosurgery related to SDM.

## Results

### Search results

A total of 639 unique articles were found through database searching and reference lists. After screening articles by title, 369 articles were excluded for the following reasons: age range of 17 years and younger, SDM outside of neurosurgery, not assessing SDM and not original articles. Of the remaining 270 articles, a further 228 articles were excluded after screening of the abstract. Finally, a total of 42 articles were assessed in full text for eligibility, whereof 18 studies were excluded due to not assessing SDM and 2 studies for not being original articles. This resulted in the inclusion of 22 studies: 14 studies identified through literature searches [2, 6, 16, 23, 25, 28, 29, 32, 35, 39, 51–53, 57] and 8 articles through screening of reference lists [1, 5, 11, 22, 34, 42, 43, 54] (see Tables 1 and 2, respectively and see the PRISMA flowchart for more information (Fig. 1)).

Of the 22 articles included, 7 focused on SDM in patients with vestibular schwannomas [6, 16, 28, 29, 34, 35, 39], 6 involved patients undergoing spinal surgery (lumbar herniated disk, lumbar spinal stenosis, spinal stenosis) [1, 2, 22, 32, 42, 54], and 4 included patients with gliomas [5, 11, 25, 57]. The remaining articles concerned brain metastases, benign brain lesions, Parkinson’s disease, evaluation of neurosurgical care, and one case report on cervical spinal stenosis. More than 4000 patients and participants were included in these articles.

We observed a heterogeneity in the methods used for the included articles. Thirteen articles were prospective with inclusion prior to treatment or at first consultation. In 15 studies, questionnaires were used and interviews were performed in 6 studies. The timing of questionnaire administration differed, ranging from before consultation, right after consultation/intervention to follow-up up to 3 years after first consultation/intervention.

Three main themes were identified:

I. Evaluation/identification of factors that influence patients’ decisions;
II. Evaluation of SDM intervention effects; and
III. Evaluation of SDM aids.

#### Evaluation/identification of factors that influence patients’ decisions

Factors influencing patients’ decisions include the perceptions and expectations of a total of 3127 patients over 14 articles [5, 6, 11, 22, 23, 28, 29, 34, 35, 42, 43, 53, 54, 57]. Methods used to evaluate the SDM process were questionnaires in 11, semi-structured interviews in two, and one study used focus groups.

The diagnosis included in these discussions was vestibular schwannoma, lumbar spinal stenosis, Parkinson’s disease, glioma, benign brain tumors, arteriovenous malformations, unruptured aneurysms, or brain metastases. Topics addressed were conservative treatment versus surgical treatment, “awake” methods versus “asleep” methods, stereotactic radiosurgery (SRS) versus SRS plus whole-brain radiotherapy, and the clinical dilemma of a trade-off between neurological function and survival time.

#### Evaluation of SDM intervention effects

The articles evaluating SDM intervention effects reflected the degree of SDM involvement for a total of 1141 patients over 6 articles [1, 16, 25, 32, 39, 51]. The diagnosis reported was glioma (84 patients), vestibular schwannoma (660 patients), lumbar disk herniation (39 patients), cervical spinal stenosis (1 patient), or any unspecified neurosurgery-related patient group (364 patients). The methodologies presented in these articles made use of questionnaires such as Hospital Anxiety and Depression Scale (HADS), short form-36 (SF-36) measuring quality of life, Pain Disability Index (PDI), Beck Depression Inventory (BDI), and questionnaires made for their study aim. One study was a case report.

Decision topics addressed by the SDM process were mainly conservative treatment versus surgical treatment or radiotherapy and the risks of surgery. Furthermore, the type of results reported included successful and mixed intervention outcomes. Successful SDM interventions reported high levels of patient involvement related to equal levels of patient satisfaction with the provided care (Tables 1 and 2). In contrast, mixed intervention outcomes were signaled by deficits in the quantity of SDM interventions being exercised. The instruments used to assess the degree of SDM included mostly questionnaires.

#### Evaluation of SDM aids

SDM aids were directly discussed for the diagnosis lumbar disk herniation (270 patients) and glioma (11 patients) in three articles [1, 2, 52]. The methodologies employed made use of structured interviews, semi-structured interviews, and questionnaires. One of the articles aimed to evaluate SDM aids and factors that influence patients’ decisions. Decision topics addressed by the SDM related to the SDM aids were not at the center of the discussion. However, SDM aids such as decision boards, video disks, and tumor 3D models were mainly found to require further testing to assess their effectivity. The results reported in these articles regarded the levels of satisfaction, barriers, and facilitators regarding the use of such SDM aids (Tables 1 and 2).

## Discussion

In this scoping review we present the current literature regarding SDM in neurosurgery. The limited extent of SDM use in the neurosurgical field was notable, and conditions more commonly included were spinal disorders and vestibular schwannomas. A wide range of methods were used, but the application of questionnaires dominated.

### Design and characteristics of included studies

There was a wide variety of different methods used in the included studies, from prospective studies with follow-up questionnaires to more explorative studies with 3D models, suggesting the lack of common methods to evaluate SDM. Although designs differed, the common aim of evaluating and incorporating SDM was present in all articles. There was a recurring theme of shortfall of information in both preoperative and postoperative settings. Some articles raise concerns that not all treatment options were presented, or that the side effects of the treatment options were not presented [29]. For the patients to be able to participate in decision-making, all the different treatment options with benefits and risks should be offered to the patient.

### Practical application of SDM

Many healthcare professionals in different medical fields agree that SDM is important for the patient when making a decision, but the practical application of SDM may be more challenging [19, 50]. Different decisional aids have been used for facilitating SDM with the patients, although the methods used seem to be unique for each article. van de Belt et al. investigated a 3D-printed model of the glioma, Zeng et al. used a decision board illustrating differences between methods and including a summary, the study by Barrett et al. used a video program for the patient to watch, and finally Andersen et al. used a paper leaflet with relevant information [1, 2, 52, 57]. The decisional aids presented have not been validated and further investigation is warranted.

Andersen et al. developed a patient decision aid to better facilitate and support SDM, a process which otherwise can be challenging [1]. Their patient decision aid was a paper leaflet with information regarding advantages and disadvantages with each surgical and non-surgical option offered, treatment outcomes, how symptoms may affect the patient and rate of severe complications after surgery. A decisional aid like the one developed by Andersen and co-authors covers the important steps in the SDM process, while also providing the patient with information that might be overlooked or considered less important by the surgeon [1].

It has been discussed that cognitive impairment associated with the tumor may cause difficulties in SDM for patients with brain tumors [20, 38]. Hewins et al. published a review on the effects of brain tumors on patients’ decision-making capacity, an important aspect in the process of SDM [20]. They concluded that the capacity for consenting to medical treatment in patients with brain tumors may need additional assessment of cognitive abilities to test the ability to consent for both treatment and research. In these patients, the support of relatives is important, and information regarding possible treatment options is also of high relevance to relatives, who often feel their needs are unmet regarding communication and information [13, 44, 48]. Involving patients and relatives more in the care may increase the understanding and can perhaps lead to better treatment compliance and overall well-being.

### Neurooncology

The articles in the field of neurooncology range from more biologically benign lesions to high-grade gliomas [43, 52]. Vestibular schwannoma was the most common tumor in which SDM was used in the decision-making process [16, 28, 29, 35], perhaps due to the different treatment modalities available (radiosurgery, surgery, radiotherapy, and wait-and-scan) [15, 43]. The treatment of vestibular schwannoma is associated with specific risks and selection of the optimal modality is a careful process [4, 55].

In the study by Moshtaghi et al., the authors sent out surveys to patients diagnosed with vestibular schwannoma and evaluated the factors that affected the decision-making process from the patient’s own perspectives [28]. Their finding included that 59% received information regarding different treatment options, and 80% visited multiple vestibular schwannoma specialists, suggesting the first visit left the patient with a feeling of uncertainty regarding their decision. The number of neurootologists consulted correlated with higher decision satisfaction. Furthermore, in additional studies, 16% of the 414 patients who underwent surgery felt pressured to select a surgical treatment for their vestibular schwannoma [28]. In an additional study, 69% of the patients only received information regarding one treatment option, mainly surgery, and usually not enough information regarding side effects of the treatments [29]. In the study by Graham et al., 20% of patients with vestibular schwannoma experienced decisional conflict and involving patients in decision-making reduced the degree of uncertainty [16]. The lack of information in an early stage may lead to waste of healthcare resources by patients seeking confirmation from multiple specialists for the same issue. Perhaps the lack of information can be improved by decisional aids to fill the information gap and fully inform the patients about possible treatment options and risks associated with the options presented.

When further exploring SDM in the field of neurooncology, it seems that most patients take an active role if information is presented adequately, as presented by Zeng et al. [57]. They illustrate how to include patients with brain metastases in a patient-centered approach where a key element is the use of comprehensible information. When the patients were presented with clear information, they could decide accordingly what was important for them.

Brennum et al. challenged the established Hippocratic principle of “primo non nocere” in favor of maximal resection and survival [5]. The participating experts and patients discussed the balance between neurological function and longer survival and found that offering more extensive surgery could be ethically acceptable. Although, even informed patients accepting neurological deficit for the benefit of longer survival may regret their decision if the outcome with neurological deficit is difficult to comprehend. The risk that a patient misunderstands the surgeon is a risk with surgery beyond maximal safe resection, as they most often lack the experience of neurological deficit and may perhaps idolize the difficult decision they face [31]. Still, a more person-centered care where the patient is considered a partner in the decision-making process may improve health outcomes and increase patient satisfaction [12].

### Strengths and limitations

The wide spectrum of approaches to SDM may indicate that implementation of SDM is challenging. In this study, we included a variety articles to provide a thorough update of the use of SDM in neurosurgery. Although our methodology followed a broad approach, we found a limited number of studies, a large methodological variability between studies, and a variable sample size in the selected studies, indicating that SDM is still in its infancy in neurosurgery. Additionally, we identified eight articles through references suggesting some keywords were not covered by the search blocks. This may be due to the fact that most of the articles (7) identified through references discussed topics related to *factors that influence patients’ decisions* and were not aimed to primarily assess the effects of decision-making processes. Furthermore, there may be more studies that explored the topic of SDM peripherally, or through use of proxies, that escaped the scope of our search.

## Conclusion

Shared decision-making is a tool to involve patients in the decision-making process, to provide optimal care also considering patients preferences, and to include what they feel is important in the decision process. This review illustrates the relative lack of SDM in the neurosurgical literature and can hopefully serve as useful information regarding SDM and be used as a foundation to better involve neurosurgical patients in the decision-making process. Although the results provided indicate that there may be a potential benefit of using SDM, to what extent and how SDM influences treatment provided, outcome, and patient satisfaction remains to be seen. Finally, the use of decision aids may be a meaningful contribution to the SDM process.

## Supplementary Information

Below is the link to the electronic supplementary material.

Supplementary file1 (DOCX 16 KB)
