# Supplementary material for: Shared decision-making in neurosurgery: a scoping review
Source: Acta Neurochir (Wien). 2021 May 3;163(9):2371–82. doi: 10.1007/s00701-021-04867-3 (PMC8357744; doi:10.1007/s00701-021-04867-3)
Supplement: Supplementary file 1 — Supplementary file1 (DOCX 16 KB) [file 701_2021_4867_MOESM1_ESM.docx]

**Supplementary table 1**. Detailed search strategy in PubMed (Conducted 21-02-16, retrieved 392 articles).

| Search | Search term |
| --- | --- |
| 1 | (Neurosurgery[mesh] OR neurosurgery[tiab] OR neurosurgeries[tiab] OR neurosurgical[tiab] OR neuro-oncology surgery[tiab] OR neuro-oncology surgeries[tiab] OR neurologic surgical[tiab] OR neurological surgery[tiab] OR spinal surgery[tiab] OR glioma[mesh] OR gliomas[tiab] OR glioma[tiab] OR Meningioma[mesh] OR Meningioma[tiab] OR Meningiomas[tiab] OR Schwannoma[tiab] OR Schwannomas[tiab] OR Pituitary Neoplasms[mesh] OR  Pituitary Neoplasms[tiab] OR Pituitary adenomas[tiab] OR Pituitary tumors[tiab] OR Pituitary adenoma[tiab] OR Pituitary tumor[tiab] OR Pituitary tumours[tiab] OR Pituitary tumour[tiab]) |
| 2 | (Person centered[tiab] OR person centred[tiab] OR person-centered[tiab] OR person-centred[tiab] OR person centeredness[tiab] OR personcenteredness[tiab] OR person centredness[tiab] OR client-centered[tiab] OR client-centred[tiab] OR client centered[tiab] OR client centred[tiab] OR client centeredness[tiab] OR clientcenteredness[tiab] OR client centredness[tiab] OR patient centered[tiab] OR patient centred[tiab] OR patient-centered[tiab] OR patient-centred[tiab] OR patient centeredness[tiab] OR patientcenteredness[tiab] OR patient centredness[tiab] OR relationship-centered[tiab] OR relationship-centred[tiab] OR client perspective[tiab] OR client perspectives[tiab] OR patient involvement[tiab] OR patient participation[tiab] OR Patient-Centered Care [Mesh] OR Person-centered therapy[mesh] OR narrative medicine[mesh] OR narrative medicine[tiab] OR shared decision[tiab] OR Decision Making, Shared[mesh] OR patient preference[mesh] OR patient preference[tiab] OR patient preferences[tiab] OR patient focused[tiab] OR person focused[tiab] OR patient-focused[tiab] OR person-focused[tiab] OR relationship centeredness[tiab] OR relationshipcenteredness[tiab] OR relationship centredness[tiab] OR women centeredness[tiab] OR womencenteredness[tiab] OR women centredness[tiab] OR family centeredness[tiab] OR familycenteredness[tiab] OR family centredness[tiab] OR people centeredness[tiab] OR peoplecenteredness[tiab] OR people centredness[tiab]) |
| 3 | #1 AND #2  Limited to publication years 2000-2021, English |

**Supplementary table 2**. Detailed search strategy in Scopus (Conducted 21-02-16, retrieved 481 articles)

| Search | Search term |
| --- | --- |
| 1 | TITLE-ABS-KEY ( neurosurgery OR neurosurgery OR neurosurgeries OR neurosurgical OR "neuro-oncology surgery" OR "neuro-oncology surgeries" OR "neurologic surgical" OR "neurological surgery" OR "spinal surgery" OR glioma OR gliomas OR glioma OR meningioma OR meningioma OR meningiomas OR schwannoma OR schwannomas OR "Pituitary Neoplasms" OR "Pituitary Neoplasms" OR "Pituitary adenomas" OR "Pituitary tumors" OR "Pituitary adenoma" OR "Pituitary tumor" OR "Pituitary tumours" OR "Pituitary tumour" ) |
| 2 | TITLE-ABS-KEY ( "Person centered" OR "person centred" OR person-centered OR person-centred OR "person centeredness" OR personcenteredness OR "person centredness" OR client-centered OR client-centred OR "client centered" OR "client centred" OR "client centeredness" OR clientcenteredness OR "client centredness" OR "patient centered" OR "patient centred" OR patient-centered OR patient-centred OR "patient centeredness" OR patientcenteredness OR "patient centredness" OR relationship-centered OR relationship-centred OR "client perspective" OR "client perspectives" OR "patient involvement" OR "patient participation" OR "Patient-Centered Care" OR "Person-centered therapy" OR "narrative medicine" OR "narrative medicine" OR "shared decision" OR "patient preference" OR "patient preference" OR "patient preferences" OR "patient focused" OR "person focused" OR patient-focused OR person-focused OR "relationship centeredness" OR relationshipcenteredness OR "relationship centredness" OR "women centeredness" OR womencenteredness OR "women centeredness" OR "family centeredness" OR familycenteredness OR "family centeredness" OR "people centeredness" OR peoplecenteredness OR "people centeredness" ) |
| 3 | #1 AND #2  Limited to publication years 2000-2021, English |
